# Genome Size Variation among and within *Camellia* Species by Using Flow Cytometric Analysis

**DOI:** 10.1371/journal.pone.0064981

**Published:** 2013-05-27

**Authors:** Hui Huang, Yan Tong, Qun-Jie Zhang, Li-Zhi Gao

**Affiliations:** 1 Key Laboratory of Biodiversity and Biogeography, Kunming Institute of Botany, Chinese Academy of Sciences, Kunming, China; 2 Plant Germplasm and Genomics Center, Germplasm Bank of Wild Species in Southwest China, Kunming Institute of Botany, Chinese Academy of Sciences, Kunming, China; 3 University of the Chinese Academy of Sciences, Beijing, China; University of Massachusetts, United States of America

## Abstract

**Background:**

The genus *Camellia*, belonging to the family Theaceae, is economically important group in flowering plants. Frequent interspecific hybridization together with polyploidization has made them become taxonomically “difficult taxa”. The DNA content is often used to measure genome size variation and has largely advanced our understanding of plant evolution and genome variation. The goals of this study were to investigate patterns of interspecific and intraspecific variation of DNA contents and further explore genome size evolution in a phylogenetic context of the genus.

**Methodology/Principal Findings:**

The DNA amount in the genus was determined by using propidium iodide flow cytometry analysis for a total of 139 individual plants representing almost all sections of the two subgenera, *Camellia* and *Thea*. An improved WPB buffer was proven to be suitable for the *Camellia* species, which was able to counteract the negative effects of secondary metabolite and generated high-quality results with low coefficient of variation values (CV) <5%. Our results showed trivial effects on different tissues of flowers, leaves and buds as well as cytosolic compounds on the estimation of DNA amount. The DNA content of *C. sinensis* var. *assamica* was estimated to be 1C = 3.01 pg by flow cytometric analysis, which is equal to a genome size of about 2940 Mb.

**Conclusion:**

Intraspecific and interspecific variations were observed in the genus *Camellia*, and as expected, the latter was larger than the former. Our study suggests a directional trend of increasing genome size in the genus *Camellia* probably owing to the frequent polyploidization events.

## Introduction

The genome size is the amount of DNA in an unreplicated, basic, gametic chromosome set [1]. The study on genome size variation often provides a strong unifying element in biology with practical and predictive uses. Myriad organismal and ecological traits are frequently associated with the variation in genome size [2], [3], [4]. Therefore, the measurement of the DNA content and genome size is often employed to better understand plant evolution and enhance comparative analyses of genome evolution [5].

Genome size variation among angiosperms nearly 2400-fold, ranging from 1C = 0.06 pg in *Genlisea margaretae* to 1C = 152.23 pg in the *Paris japonica* [6], with an extensive variation occurring even within groups. The average within-genus size variation is 3-fold, with an upper bound of more than 63-fold [7]. Indeed, intraspecific variation in genome size has also been observed in many plants [8], [9]. The observed 37% variation in DNA content was found to be correlated with the number and size of heterochromatic knobs in *Zea mays* [10]. Another example is DNA content of flax, *Linum usitatissimum*, which may vary within a single generation when the plants are grown under specific environmental conditions [11]. However, Greilhuber [12] suggested that earlier numerous reports of genome size variation below the species level were dismissed by inaccurate methods which lead to the unreliable measurement results, as clearly shown in studies on endogenous staining inhibitors [13], [14], [15]. Moreover, a great stability of the nuclear genome size has been reported in geographically isolated populations of *Sesleria albicans* [16], different species of *Settaria* [17], *Cistus* [18], *Capsicum* [19], and diverse cultivars of pea and onion [20], [21]. Nevertheless, these findings should instantly provoke the question whether it is a real variation in DNA amount or simply an artifact of intraspecific variation in genome size.

The relative frequency of increases and decreases in DNA content still remains unresolved in angiosperm phylogeny [22]. Besides polyploidization, genome size is primarily influenced by the proportion of non-genic repetitive DNA, much of which originates from transposable elements [23], [24]. In particular, copy number of retrotransposons may dramatically vary from one to another genome [25], [26]. An increase in genome size may result from the amplification and accumulation of retrotransposons. Nevertheless, the decrease in genome size can be caused by a higher overall rate of deletions than insertions, selection against transposable elements, unequal crossing over, and illegitimate recombination [27]. The occurrence and extent of genome size variation among and within plant species as well as evolutionary mechanisms behind still remain controversial and more investigations are fairly needed.

The genus *Camellia* has been long attracted considerable attention due to its greatly economic values, broadly geographic distribution and remarkable species diversity. The main economic value of *Camellia* is the production of tea made from the young leaves of *C. sinensis* var. *sinensis* and *C. sinensis* var. *assamica*. In addition, *C. oleifera* has been primarily used for cooking oil extracted from seeds [28]. Besides, *Camellia* species are of great ornamental values especially represented by *C. japonica*, *C. reticulata* and *C. sasanqua*. The genus is taxonomically ranked as one of the most challengingly difficult taxa in plants, whose complexity is primarily governed by frequent hybridization, accompanied by polyploidization and subsequent stabilization of novel forms by clonal growth [29]. The classification of species using a morphology-based system is often changeable and also disputed based on chromosome pairing behavior of hybrids [30]. As a result, the boundaries between taxa of various ranks are still a subject of dispute. According to Chang et al. [31], *Camellia* was classified into a total of 18 sections of four subgenera, which approximately comprised 361 species. However, Min et al. [28] taxonomically classified the genus into 14 sections of two subgenera, consisting of only about 120 species. The available sequence-based phylogeny of this genus is necessarily limited, and many controversies have long existed with regard to their taxonomical classification. The nuclear DNA content is in some cases useful as a supportive marker for a reliable delineation of problematic taxa and possesses a predictive value to infer evolutionary relationships [32]. Unfortunately, the lack of nuclear DNA contents apparently prevents us from understanding the diversification and evolution of the *Camellia* species. The knowledge of interspecific and intraspecific patterns of genome size variation may help to enlighten the evolution and particularly the involved evolutionary events such as hybridization and polyploidization in the genus. In the present study, we estimated genome size of *C. sinensis* var. *assamica* by using flow cytometric analysis. In the hope of better understanding the diversification and evolution in the genus *Camellia*, we extensively investigated interspecific and intraspecific patterns of DNA content variation in representative sections and species. The data presented here are intended to fill a gap that exists in the current genomic knowledge base of *Camellia* and take nuclear DNA content variation as a useful marker to predict and infer evolutionary relationships in such problematic taxa.

## Materials and Methods

### Plant materials

Materials of the *Camellia* plants used in this study were kindly provided by Kunming Institute of Botany (Chinese Academy of Sciences), Tea Research Institute (Yunnan Academy of Agricultural Sciences, China) and International *Camellia* Species Garden (Jinhua, Zhejiang, China) from May to July of 2010. All necessary permits were obtained for the described field studies; names of the persons or authority who issued the permission for each location are as below: Wei-bang Sun, Kunming Botanical Garden, Chinese Academy of Sciences; Ming-zhi Liang, Tea Research Institute, Yunnan Agricultural Academy of Sciences, Yunnan, China; Ji-yuan Li, International *Camellia* Species Garden, Jinhua, Zhejiang, China. We collected flowers, leaves and buds from field-growing trees, which were either analyzed immediately or maintained in a refrigerator on moistened paper for a maximum of two days until use. Considering many controversies of the genus *Camellia*, the collected plant materials were classified and analyzed by using two taxonomical treatments (Min taxonomic system: MTS; Chang and Ren taxonomic system: CRTS) [28], [31] in hope of the delineation of problematic taxa based on nuclear DNA contents.

### Sample preparation

Approximately 40–50 mg of flowers, leaves and buds were separately used for the sample preparation. Nuclei suspensions were improved according to Galbraith et al. [33] and WPB isolation buffers [34], including 0.2 mM Tris.HCl, 4 mM MgCl_2_.6H_2_O, 2 mM EDTA Na_2_.2H_2_O, 86 mM NaCl, 2.0 mM dithiothreitol (Sigma-Aldrich CHIEMIE Gmbh, Steinheim, Germany), 1% (w/v) PVP-10, 1% (v/v) Triton X-100, (pH 7.5). For each case, 1 mL of ice-cold nuclei suspensions was added to a Petri dish containing the plant tissue, which was chopped using a sharp razor blade. The resulting homogenate was filtered through a 50-µm nylon filter to remove cell fragments and large debris. Nuclei were treated with 50 µg mL^−1^ RNase (Fluka, Buchs, Switzerland) and stained with 50 µg mL^−1^ propidium iodide (PI) (Sigma, St. Louis, MO, USA). The samples were kept on ice until further uses. Maize (*Z. mays* L. cv. B73) with a DNA content of 1C = 2.35 pg, namely 2300 Mb [35], was employed as a standard.

### Flow cytometry measurements

Nuclear samples were analyzed by using a BD FACSCalibur (USA) flow cytometer. The instrument was equipped with an air-cooled argon-ion laser tuned at 15 mW and operating at 488 nm. PI fluorescence was collected through a 645-nm dichroic long-pass filter and a 620-nm band-pass filter. The amplifier system was set to a constant voltage and gained throughout the experiments. Usually, 10,000 nuclei were analyzed for each sample. The results of flow cytometry were further analyzed by using the Cellquest software and gated to selectively visualize all cells of interest which gather densely in dotplot map while eliminating results from unwanted particles. Here, CV = D/M×l00%, D is the standard deviation of the cell distribution and M is the average of cell distribution. The average of coefficient of variation values (CV) was used to evaluate the results with which CV<5% were considered as reliable. Nuclear DNA content was calculated as a linear relationship between the ratio of 2C-value peaks of the sample and standard.

### Tests for inhibitors

To determine the impact of secondary metabolites on the fluorescence of nuclei, we tested the unidentified compounds in leaves of *C. sinensis* var. *assamica* cv. yunkangshihao that reduce PI fluorescence of maize (*Z. mays* L. cv. B73) nuclei as follows. Treatment A consisted of PI-stained nuclei from the independently processed and stained 20–25 mg leaves of *C. sinensis* var. *assamica* and *Z. mays*, respectively. *C. sinensis* var. *assamica* and *Z. mays* were simultaneously processed (co-chopped) and stained with PI, called as treatment B. After staining, these samples were individually measured for mean PI fluorescence, and the experiment was replicated for a total of three times. The fluorescence of nuclei from leaves of the marker simultaneously processed with *C. sinensis* var. *assamica* materials was compared with that from independently processed leaves of the marker and gave evidence of inhibitors.

### Statistical analyses

Differences and correlations among variables between the *Camellia* species as well as different tissues were statistically tested using one-way ANOVA implemented with the software SPSS (SigmaStat for Windows Version 3.1, SPSS Inc., Richmond, CA, USA).

## Results

### Optimization of DNA flow cytometry for the *Camellia* species

**Figure 1:**
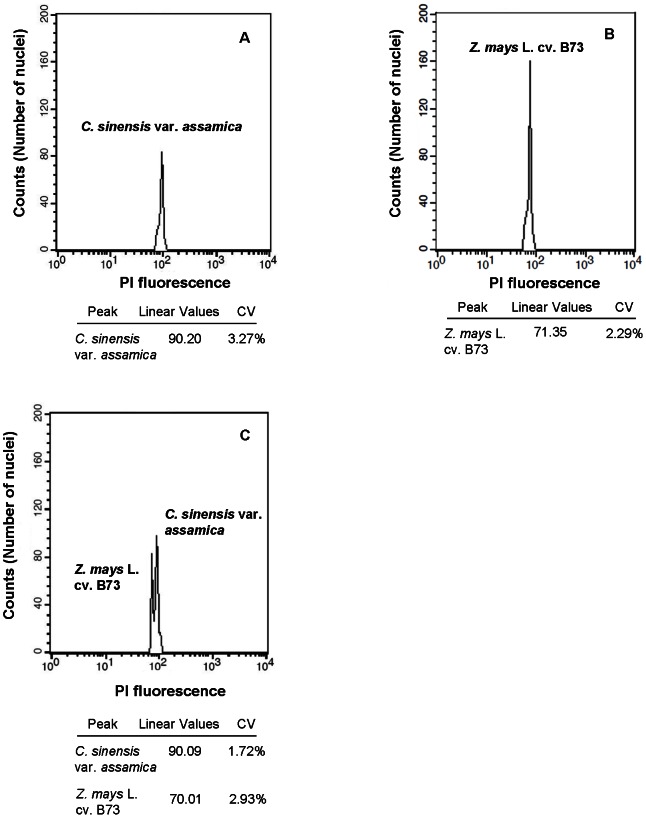
Cytogram of fluorescence intensity of *C. sinensis* var. *assamica* and *Z. mays* L. cv. B73 nuclei isolated with an improved WPB buffer. Leaves of *C. sinensis* var. *assamica* and *Z. mays* that were treated individually (a, b) or simultaneously processed (co-chopped) (c), and stained with PI. X: Relative fluorescence; Y: Number of nuclei.

In this study, a total of five nuclear isolation buffers were compared, which included Galbraith [33], LB01 [36], Otto [37], [38], Tris.MgCl_2_ [39], and WPB [34] (data not shown). An improved WPB isolation buffer was finally chosen and employed in the flow cytometry, which was able to counteract the negative effects of tannic acid better than the other four buffers [40], [41]. The optimization of DNA flow cytometry generated high-quality results with low CV<5% in the present study. To determine a suitable plant tissue for the flow cytometry analysis for the *Camellia* species, we sampled and detected a total of three tissues, including flowers, leaves and buds from the eight species, representing up to five sections of the genus, *C. oleifera*, *C. pyxidiacea* var. *rubituberculata*, *C. impressinervis*, *C. grijsii* var. *grijsii*, *C. reticulata* (cv. honghuayoucha and cv. zipao), *C. editha*, and *C. japonica* (cv. feilipu). The nuclear DNA contents of *Camellia* species were presented as picograms and the variability of 2C-values among different tissues from a single specie was tested using one-way ANOVA (Table 1). Our results showed that 2C-values of the three tissues from a single *Camellia* plant had no significant differences between each other (*P*>0.05). The estimation of 2C-values, taking *C. impressinervis* for example, were 4.56±0.167, 4.59±0.138 and 4.61±0.161 pg for flower, leave and bud, respectively (*P* = 0.925>0.05). The largest discrepancy (0.13 pg/2C) between 2C-values of the three tissues were observed in *C. editha*, with 2C-value of 5.65±0.123 pg in flowers and 5.52±0.409 pg in leaves, respectively (*P* = 0.782>0.05). The standard deviation (SD) of 2C-value of three tissues from a single plant was more evident in the species with a large genome than the species with a small genome. For example, *C. oleifera* with the highest SD (0.691) in flowers and buds had the average 2C-value of 17.47 pg, while 2C-values of the three tissues of *C. oleifera* had no significant differences between each other (*P*>0.05). In addition, results showed that the flower color pigments had no obvious influence on staining results (Table 1). In order to test the impact of cytosolic compounds on the fluorescence of *Camellia* nuclei, we further measured and compared two filtrates of *C. sinensis* var. *assamica* cv. yunkangshihao (Fig. 1a) and *Z. mays* L. cv. B73 (Fig. 1b) which were treated individually, with a mixed filtrate which was co-chopped together (Fig. 1c). The PI fluorescence (linear values) of *C. sinensis* var. *assamica* and *Z. mays* was 90.20 and 71.35, respectively, when they were individually treated. In the co-chopped treatment, the PI fluorescence (linear values) for these two species was 90.09 and 70.01, respectively, with lower intensity peaks compared with the former. There existed 0.11 and 1.34 differences of PI fluorescence between samples treated individually and simultaneously. The average of CV were 3.27% and 2.29% for *C. sinensis* var. *assamica* (Fig. 1a) and *Z. mays* (Fig. 1b) alone, while the average of CV were 1.72% and 2.93% for them (Fig. 1c), respectively, which were simultaneously processed and stained.

**Table 1:** Comparisons of nuclear DNA amount (2C, pg) estimated with flow cytometry in different tissues of the eight species in the genus *Camellia*.

Species (Min et al. 2010) [28]	Species (Chang and Ren, 1998) [31]	Flower	SD	Leaf	SD	Bud	SD	Flower colors	*P*
**sect. ** ***Paracamellia***	**sect. ** ***Oleifera***								
***C. oleifera***	***C. oleifera***	17.53	0.691	17.46	0.213	17.41	0.691	White	0.968
**sect. ** ***Tuberculata***	**sect. ** ***Tuberculata***								
*C. pyxidiacea* var. *rubituberculata*	*C. rubituberculata*	4.56	0.245	4.57	0.112	4.63	0.113	Red	0.863
**sect. ** ***Archecamellia***	**sect. ** ***Chrysantha***								
*C. impressinervis*	*C. impressinervis*	4.56	0.167	4.59	0.138	4.61	0.161	Yellow	0.925
**sect. ** ***Paracamellia***	**sect. ** ***Paracamellia***								
*C. grijsii* var. *grijsii*	*C. yuhsienensis*	15.24	0.530	15.22	0.27	15.21	0.330	White	0.996
**sect. ** ***Camellia***	**sect. ** ***Camellia***								
*C. reticulata* cv. honghuayoucha	*C. reticulata* cv. honghuayoucha	15.31	0.339	15.37	0.015	15.33	0.550	Light red	0.980
**sect. ** ***Camellia***	**sect. ** ***Camellia***								
*C. reticulata* cv. zipao	*C. reticulata* cv. zipao	15.04	0.254	15.14	0.285	15.13	0.381	Dark red	0.911
**sect. ** ***Camellia***	**sect. ** ***Camellia***								
*C. edithae*	*C. edithae*	5.65	0.123	5.52	0.409	5.64	0.037	Red	0.782
**sect. ** ***Camellia***	**sect. ** ***Camellia***								
*C. japonica* cv. feilipu	*C. japonica* cv. feilipu	5.72	0.023	5.81	0.264	5.75	0.155	Pink	0.824

*Z. mays* L. cv. B73 was employed as a standard. The colors of flowers are given in the Table. All materials were collected from Kunming Institute of Botany, Chinese Academy of Sciences (KIBCAS).

### Intraspecific genome size variation within *C. sinensis* var. *assamica*

**Figure 2:**
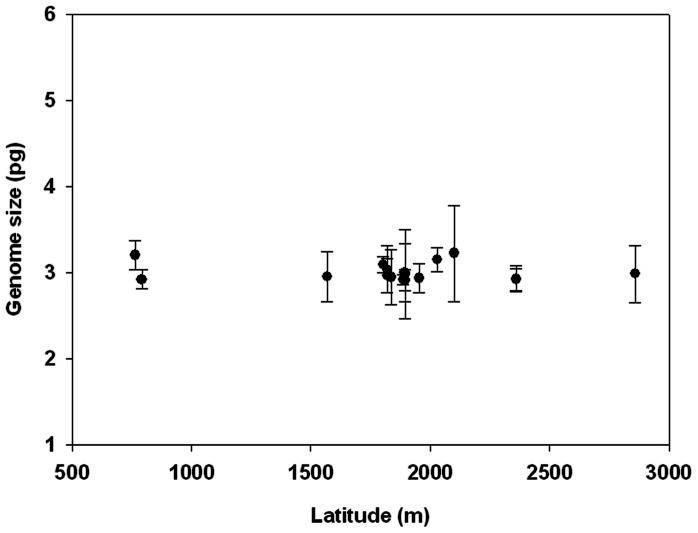
The relationship between genome size (pg) and latitudinal origins of 17 cultivars of *C. sinensis* var. *assamica*.

To determine the extent and patterns of intraspecific nuclear DNA content variation, we sampled a total of 17 cultivars of *C. sinensis* var. *assamica*, which extensively represent different geographic and ecological origins of the species in Yunnan Province, China (Table 2). The 2C DNA content varied only 1.1-fold among different cultivars from 5.82±0.119 pg in *C. sinensis* var. *assamica* cv. zijuan to 6.45±0.559 pg in *C. sinensis* var. *assamica* cv. manghui, with a standard deviation of 0.20. Based on the mean DNA content of all the measured cultivars (1C = 3.01 pg), the genome size of *C. sinensis* var. *assamica* was estimated to be 2940 Mb by using 1 pg DNA = 978 Mb [42]. To determine the relationship between latitudes and DNA contents of those measured *C. sinensis* var. *assamica* cultivars, we further performed the regression analysis of them. The results exhibited an *R^2^* value of 0.033 and a low slope value of -7.418e-5, which was not statistically different from zero (Fig. 2).

**Table 2:** Nuclear DNA amount of *C. sinensis* var. *assamica* cultivars estimated with flow cytometry.

Species	Chromosome Number (2n)	Estimation of Ploidy Levels	2C-value (pg)	SD	Latitude/Longitude
*C. sinensis* var. *assamica* cv. bijiang	30	2n = 2x	5.97	0.333	26°55′N/98°51′E
*C. sinensis* var. *assamica* cv. bingdaohei	30	2n = 2x	5.91	0.291	23°38′N/99°53′E
*C. sinensis* var. *assamica* cv. changning	30	2n = 2x	5.93	0.196	24°50′N/99°36′E
*C. sinensis* var. *assamica* cv. dasiyuantou	30	2n = 2x	5.83	0.132	24°32′N/99°55′E
*C. sinensis* var. *assamica* cv. datuan	30	2n = 2x	5.84	0.06	21°54′N/100°26′E
*C. sinensis* var. *assamica* cv. fengqing	30	2n = 2x	5.86	0.156	24°32′N/99°55′E
*C. sinensis* var. *assamica* cv. manghui	30	2n = 2x	6.45	0.559	24°25′N/100°07′E
*C. sinensis* var. *assamica* cv. manluo	30	2n = 2x	5.87	0.174	22°59′N/102°24′E
*C. sinensis* var. *assamica* cv. mengtong	30	2n = 2x	6.07	0.274	24°50′N/99°36′E
*C. sinensis* var. *assamica* cv. mengyang	30	2n = 2x	6.40	0.168	22°05′N/100°53′E
*C. sinensis* var. *assamica* cv. naka	30	2n = 2x	6.18	0.093	23°29′N/100°42′E
*C. sinensis* var. *assamica* cv. nongdaoqin	30	2n = 2x	5.84	0.108	24°00′N/97°51′E
*C. sinensis* var. *assamica* cv. tuantian	30	2n = 2x	5.89	0.321	25°02′N/98°29′E
*C. sinensis* var. *assamica* cv. xiaogude	30	2n = 2x	6.30	0.139	25°03′N/100°30′E
*C. sinensis* var. *assamica* cv. xishelu	30	2n = 2x	5.96	0.520	25°01′N/101°32′E
*C. sinensis* var. *assamica* cv. yunkangshihao	30	2n = 2x	6.00	0.333	25°02′N/102°43′E
*C. sinensis* var. *assamica* cv. zijuan	30	2n = 2x	5.82	0.119	25°02′N/102°43′E

*Z. mays* L. cv. B73 was employed as a standard. Chromosome number was taken from Min et al. (2010) [28]. All materials were collected from Tea Research Institute, Yunnan Academy of Agricultural Sciences (TRIYAAS), China. The information of latitude, longitude and altitude of germplasm origins was kindly provide by TRIYAAS.

### Interspecific genome size variation of sections *Thea* and *Camellia*

**Figure 3:**
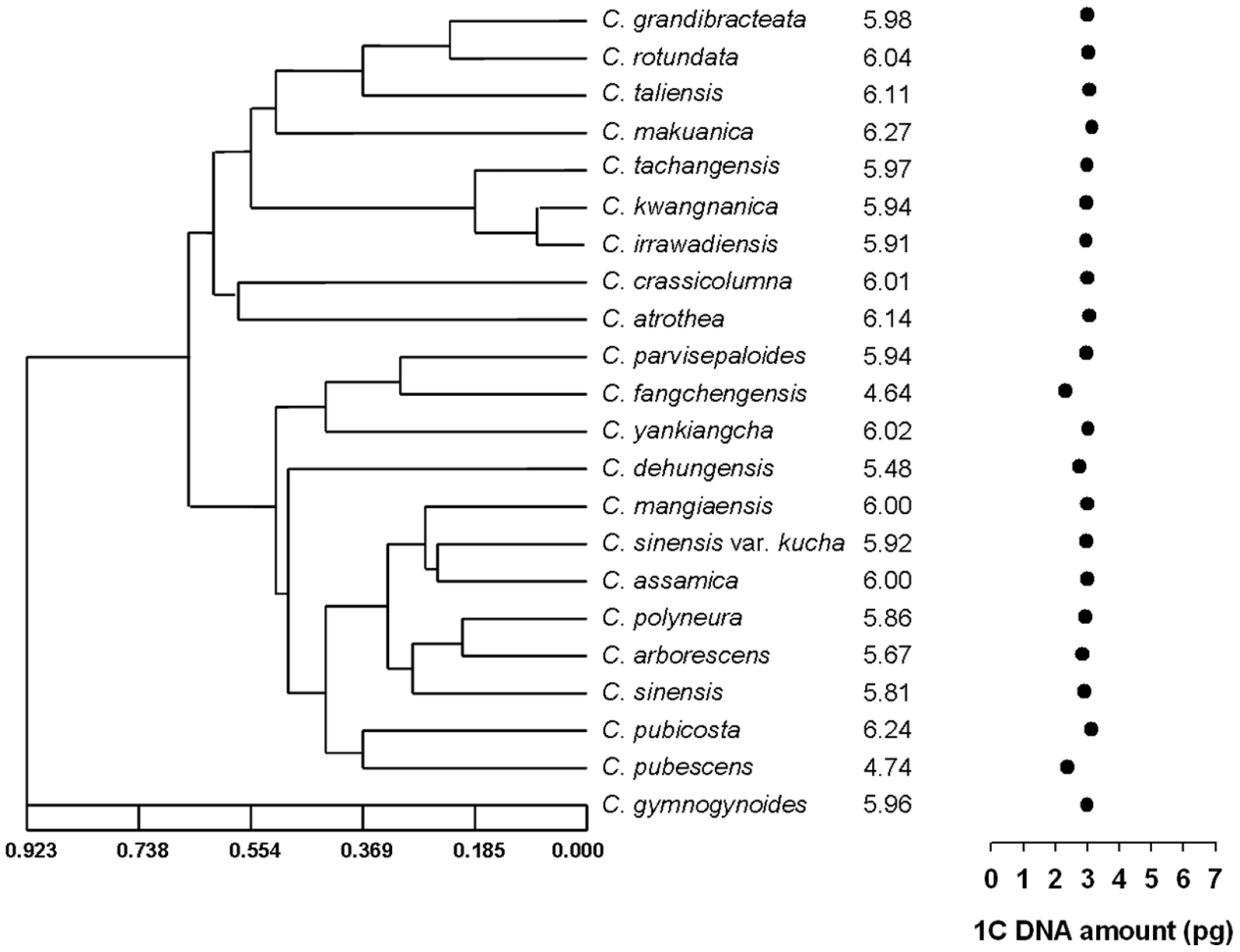
Nuclear DNA contents and evolutionary relationships among members of the section *Thea* [31]. The phylogenetic tree of the section *Thea* was constructed by using UPGMA and Nei and Li's similarity coefficient from pairwise comparisons between the 22 species and varieties based on RAPD markers [44]. The estimated 2C-values for each species are shown on the right of species, while the 1C DNA amount (pg) which also equals the genome size is shown by •.

The 2C-values of the 31 diploid species were measured in the section *Thea* [43] (Table 3). The 2C DNA contents varied 1.5-fold among these species, ranging from 4.45±0.293 pg in *C. gymnogyna* to 6.51±0.085 pg in *C. ptilophylla*. The overall mean nuclear 2C DNA content of all studied species was 5.60 pg with a 0.63 standard deviation. The DNA contents of interspecific variation (1.5-fold) in the section *Thea*, as expected, was somewhat larger than intraspecific variation (1.1-fold) among the representative cultivars of *C. sinensis* var. *assamica*. Apparently, our estimates of DNA ploidy (2n = 2x) based on DNA contents of these measured species were confirmed by conventional chromosome counting (2n = 30) (Table 3). The estimated 2C-values of the 22 species from the section *Thea* were then marked along the phylogenetic tree to show genome size variation and evolutionary relationships among species (Fig. 3). The phylogenetic tree was constructed by using UPGMA and Nei and Li's similarity coefficient from pairwise comparisons between the species based on RAPD markers [44]. In spite of slight variations, nuclear DNA contents were not randomly distributed and appeared largely conserved across the majority of the species under investigation. However, *C. fengchengensis* (4.64±0.341 pg) and *C. pubescens* (4.74±0.223 pg) were apparently found to exhibit lower DNA content than other species. Such decreased estimates of DNA content seemingly led to counterpart differences between two pairs of closely related species, *C. parvisepaloides* (5.94±0.243 pg) and *C. fengchengensis* (4.64±0.341 pg), *C. pubicosta* (6.24±0.196 pg) and *C. pubescens* (4.74±0.223 pg), with Δ 2C DNA contents of 1.3 and 1.5 pg, respectively.

**Table 3:** Nuclear DNA amount of the section *Thea* species estimated with flow cytometry.

Species (Min et al. 2010) [28]	Species (Chang and Ren, 1998) [31]	Chromosome Number (2n)	Estimation of Ploidy Levels	2C-value (pg)	SD	Origins
*C. costata*	*C. kwangtungensis*	30	2n = 2x	4.59	0.402	ICSG
*C. costata*	*C. danzaiensis*	30	2n = 2x	4.97	0.540	ICSG
*C. crassicolumna*	*C. crassicolumna*	30	2n = 2x	6.01	0.134	TRIYAAS
*C. crassicolumna* var. *crassicolumna*	*C. atrothea*	30	2n = 2x	6.14	0.188	TRIYAAS
*C. crassicolumna* var. *crassicolumna*	*C. makuanica*	30	2n = 2x	6.27	0.267	TRIYAAS
*C. crassicolumna* var. *crassicolumna*	*C. rotundata*	30	2n = 2x	6.04	0.218	TRIYAAS
*C. fangchengensis*	*C. fengchengensis*	30	2n = 2x	4.64	0.341	ICSG
*C. grandibracteata*	*C. grandibracteata*	30	2n = 2x	5.98	0.233	TRIYAAS
*C. gymnogyna*	*C. gymnogyna*	30	2n = 2x	4.45	0.293	ICSG
*C. kwangsiensis*	*C. kwangsiensis*	30	2n = 2x	5.86	0.420	ICSG
*C. kwangsiensis* var. *kwangnanica*	*C. kwangnanica*	30	2n = 2x	5.94	0.471	TRIYAAS
*C. leptophylla*	*C. leptophylla*	30	2n = 2x	4.49	0.236	ICSG
*C. ptilophylla*	*C. ptilophylla*	30	2n = 2x	6.51	0.085	ICSG
*C. ptilophylla*	*C. pubescens*	30	2n = 2x	4.74	0.223	ICSG
*C. pubicosta*	*C. pubicosta*	30	2n = 2x	6.24	0.196	TRIYAAS
*C. sinensis*	*C. sinensis*	30	2n = 2x	5.81	0.171	TRIYAAS
*C. sinensis* var. *assamica*	*C. assamica*	30	2n = 2x	6.00	0.333	TRIYAAS
*C. sinensis* var. *assamica*	*C. manglaensis*	30	2n = 2x	6.00	0.182	TRIYAAS
*C. sinensis* var. *assamica*	*C. polyneura*	30	2n = 2x	5.86	0.214	TRIYAAS
*C. sinensis* var. *assamica*	*C. sinensis* var. *kucha*	30	2n = 2x	5.92	0.185	TRIYAAS
*C. sinensis* var. *assamica*	*C. yunkiangica*	30	2n = 2x	6.02	0.233	TRIYAAS
*C. sinensis* var. *dehungensis*	*C. dehungensis*	30	2n = 2x	5.48	0.060	TRIYAAS
*C. sinensis* var. *dehungensis*	*C. parvisepaloides*	30	2n = 2x	5.94	0.243	TRIYAAS
*C. sinensis* var. *pubilimba*	*C. angustifolia*	30	2n = 2x	4.75	0.237	ICSG
*C. sinensis* var. *pubilimba*	*C. parvisepala*	30	2n = 2x	4.59	0.249	ICSG
*C. sinensis* var. *sinensis*	*C. arborescens*	30	2n = 2x	5.67	0.343	TRIYAAS
*C. tachangensis*	*C. tachangensis*	30	2n = 2x	5.97	0.009	TRIYAAS
*C. tachangensis* var. *remotiserrata*	*C. gymnogynoides*	30	2n = 2x	5.96	0.167	TRIYAAS
*C. tachangensis* var. *remotiserrata*	*C. jinyunshanica*	30	2n = 2x	4.77	0.345	ICSG
*C. taliensis*	*C. irrawadiensis*	30	2n = 2x	5.91	0.213	TRIYAAS
*C. taliensis*	*C. taliensis*	30	2n = 2x	6.11	0.108	TRIYAAS

*Z. mays* L. cv. B73 was employed as a standard. Chromosome numbers were adopted from previous studies and the index to Plant Chromosome Numbers (http://mobot.mobot.org/W2T/Search/ipch.html). ICSG: International *Camellia* Species Garden; TRIYAAS: Tea Research Institute, Yunnan Academy of Agricultural Sciences.

**Figure 4:**
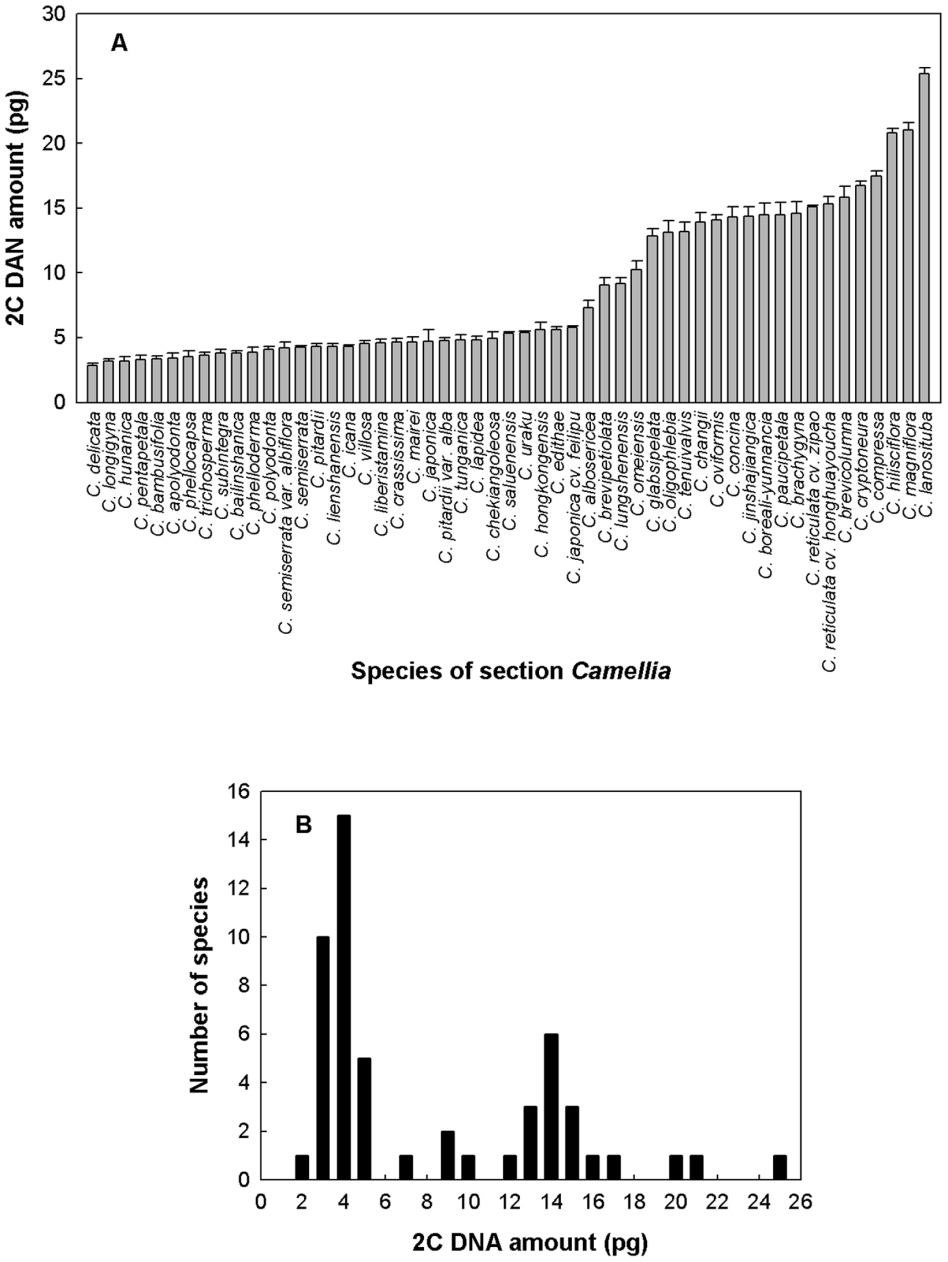
Histograms of the distribution of DNA 2C-values for the 53 species of the section *Camellia* [31]. The DNA 2C-values arranged by increasing DNA content (a) and the distribution of DNA 2C-values (b) for the 53 species of the section *Camellia*.

To investigate variations of DNA contents and polyploidy levels in the section *Camellia*, we measured 2C-values for a total of 53 species (CRTS) which were commonly recognized by the two taxonomical treatments [28], [31] (Table 4). All studied species mentioned below were followed by Chang and Ren's taxonomic system (CRTS). The 2C -values varied 8.9-fold from 2.86±0.171 pg in *C. delicata* to 25.35±0.484 pg in *C. lanosituba* (Table 4). The mean 2C-value of the section *Camellia* species was 8.61 pg, with a 5.78 standard deviation, larger than that of the section *Thea* (5.60 pg) with a 0.63 standard deviation. Figure 4a showed that the changes in DNA 2C-values of the 53 examined species arranged by increasing DNA amount in the section *Camellia*. Their 2C-values were greatly lower than 6 pg, and a small part of them were larger than 20 pg. Based on our results, these 2C-values were classified into the four groups (Group 1: <6 pg, Group 2: 6–10 pg, Group 3: 10–20 pg, and Group 4: >20 pg) (Fig. 4a, b). The 2C DNA contents of 31, 4, 15 and 3 species were found to fall into groups 1, 2, 3 and 4 with the percentages of 58.5%, 7.5%, 28.3% and 5.7%, respectively (Fig. 4b).

**Table 4:** Nuclear DNA amount of the section *Camellia* species estimated with flow cytometry.

Species (Min et al. 2010) [28]	Species (Chang and Ren, 1998) [31]	Chromosome Number (2n)	2C-value (pg)	SD	Estimation of Ploidy Levels
*C. azalea*	*C. changii*	NA*	13.92	0.718	2n = 6x
*C. chekiangoleosa*	*C. chekiangoleosa*	30	4.94	0.502	2n = 2x
*C. chekiangoleosa*	*C. crassissima*	30,90	4.63	0.289	2n = 2x
*C. chekiangoleosa*	*C. liberistamina*	30	4.59	0.304	2n = 2x
*C. concina*	*C. concina*	NA*	14.30	0.819	2n = 6x
*C. edithae*	*C. edithae*	30	5.61	0.240	2n = 2x
*C. glabsipelata*	*C. glabsipelata*	NA*	12.83	0.563	2n = 6x
*C. hongkongensis*	*C. hongkongensis*	30	5.60	0.569	2n = 2x
*C. icana*	*C. icana*	NA*	4.31	0.115	2n = 2x
*C. japonica*	*C. japonica*	30, 45	4.69	0.940	2n = 2x
*C. japonica* cv. feilipu	*C. japonica* cv. feilipu	30	5.76	0.123	2n = 2x
*C. mairei*	*C. mairei*	90	4.64	0.385	2n = 2x
*C. mairei* var. *lapidea*	*C. delicata*	60, 90	2.86	0.171	2n = 2x
*C. mairei* var. *lapidea*	*C. lanosituba*	60, 90	25.35	0.484	2n = 10x
*C. mairei* var. *lapidea*	*C. lapidea*	60	4.85	0.271	2n = 2x
*C. mairei* var. *lapidea*	*C. longigyna*	60, 90	3.19	0.171	2n = 2x
*C. mairei* var. *mairei*	*C. omeiensis*	NA*	10.23	0.664	2n = 4x
*C. mairei* var. *lapidea*	*C. phelloderma*	60	3.89	0.349	2n = 2x
*C. pitardii*	*C. pitardii*	30	4.30	0.230	2n = 2x
*C. pitardii* var. *compressa*	*C. compressa*	120	17.46	0.419	2n = 8x
*C. pitardii* var. *compressa*	*C. magniflora*	45, 90	21.04	0.561	2n = 10x
*C. pitardii* var. *cryptoneura*	*C. cryptoneura*	90	16.71	0.384	2n = 8x
*C. pitardii* var. *cryptoneura*	*C. lungshenensis*	90	9.18	0.470	2n = 4x
*C. pitardii* var. *pitardii*	*C. hunanica*	30	3.19	0.335	2n = 2x
*C. pitardii* var. *pitardii*	*C. pitardii* var. *alba*	30	4.76	0.240	2n = 2x
*C. pitardii* var. *pitardii*	*C. tunganica*	30	4.81	0.436	2n = 2x
*C. polyodonta*	*C. polyodonta*	30	4.09	0.224	2n = 2x
*C. polyodonta* var. *longicaudata*	*C. apolyodonta*	30	3.40	0.379	2n = 2x
*C. polyodonta* var. *polyodonta*	*C. oviformis*	30	14.08	0.375	2n = 6x
*C. polyodonta* var. *polyodonta*	*C. villosa*	30	4.57	0.210	2n = 2x
*C. reticulata*	*C. albosericea*	30,60,90	7.30	0.571	2n = 4x
*C. reticulata*	*C. bailinshanica*	60	3.82	0.170	2n = 2x
*C. reticulata*	*C. bambusifolia*	30	3.33	0.250	2n = 2x
*C. reticulata*	*C. boreali-yunnancia*	90	14.48	0.905	2n = 8x
*C. reticulata*	*C. brachygyna*	60	14.61	0.875	2n = 8x
*C. reticulata*	*C. brevicolumna*	90	15.85	0.824	2n = 8x
*C. reticulata*	*C. brevipetiolata*	60	9.03	0.581	2n = 4x
*C. reticulata*	*C. jinshajiangica*	90	14.38	0.725	2n = 8x
*C. reticulata*	*C. hilisciflora*	90	20.80	0.325	2n = 12x
*C. reticulata*	*C. oligophlebia*	60	13.12	0.902	2n = 6x
*C. reticulata*	*C. paucipetala*	90	14.49	0.939	2n = 8x
*C. reticulata*	*C. pentapetala*	30,60,90	3.32	0.311	2n = 2x
*C. reticulata* cv. honghuayoucha	*C. reticulata* cv. honghuayoucha	90	15.34	0.550	2n = 8x
*C. reticulata* cv. zipao	*C. reticulata* cv. zipao	90	15.10	0.094	2n = 8x
*C. saluenensis*	*C. saluenensis*	30	5.33	0.125	2n = 2x
*C. saluenensis*	*C. tenuivalvis*	30	13.19	0.712	2n = 6x
*C. semiserrata*	*C. semiserrata*	30	4.27	0.114	2n = 2x
*C. semiserrata* var. *semiserrata*	*C. phellocapsa*	30	3.51	0.441	2n = 2x
*C. semiserrata* var. *semiserrata*	*C. semiserrata* var. *albiflora*	30	4.22	0.424	2n = 2x
*C. semiserrata* var. *semiserrata*	*C. trichosperma*	30	3.62	0.235	2n = 2x
*C. subintegra*	*C. lienshanensis*	30	4.30	0.221	2n = 2x
*C. subintegra*	*C. subintegra*	30	3.80	0.269	2n = 2x
*C. uraku*	*C. uraku*	30	5.37	0.150	2n = 2x

*Z. mays* L. cv. B73 was employed as a standard. Chromosome numbers were adopted from previous studies and the index to Plant Chromosome Numbers (http://mobot.mobot.org/W2T/Search/ipch.html). All materials were collected from International *Camellia* Species Garden (ICSG).

* NA indicates that the information of chromosome number is not available.

**Figure 5:**
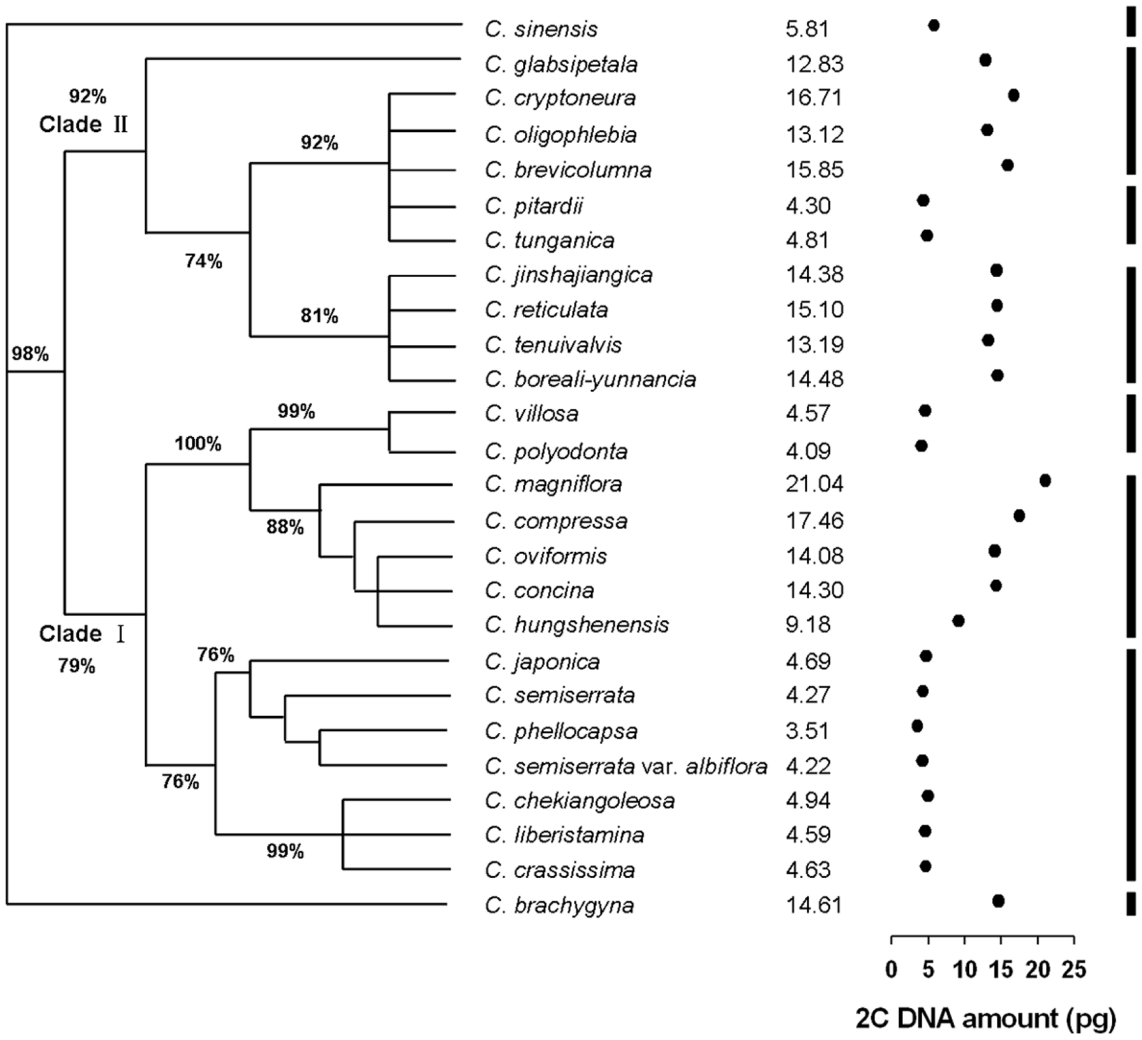
Nuclear DNA contents and evolutionary relationships among species of the section *Camellia* [31]. The phylogenetic tree was constructed based on ITS sequences [45]. The estimated 2C-values are shown on the right of each species, while the 2C DNA amount (pg) is given by • for each species.

The estimated 2C-values were then marked to the phylogenetic tree of the section *Camellia* constructed based on ITS sequences [45] (Fig. 5). The results revealed that DNA contents were mainly conserved among closely related species. Within Clade I (79%), for example, *C. japonica*, *C. semiserrata*, *C. phellocapsa*, *C. semiserrata* var. *albiflora*, *C. chekiangoleosa*, *C. liberistanmina* and *C. crassissima* closely clustered together (76%) and displayed a fairly conservation of DNA contents of approximately 3.51±0.441 pg (*C. phellocapsa*) - 4.94±0.502 pg (*C.chekiangoleosa*). Nevertheless, *C. magniflora, C. compressa, C. oviformis, C. concina* and *C. lungshenensis* clustered together (88%), but their DNA contents increased from *C. lungshenensis* (2C = 9.18±0.470 pg) to *C. magniflora* (2C = 21.04±0.561 pg). In addition, *C. polyodonta* appeared closely related with *C. villoda* (99%) and exhibited a conserved DNA content which was much smaller than the above-mentioned species within Clade I. Those species included within Clade II (92%) showed a conserved DNA content of up to 10 pg except for *C. pitardii* (2C = 4.30±0.230 pg) and *C. tunganica* (2C = 4.81±0.436 pg), which were much lower than that of other species from the same lineage.

### Genome size variation among the *Camellia* species from representative sections of the genus

**Figure 6:**
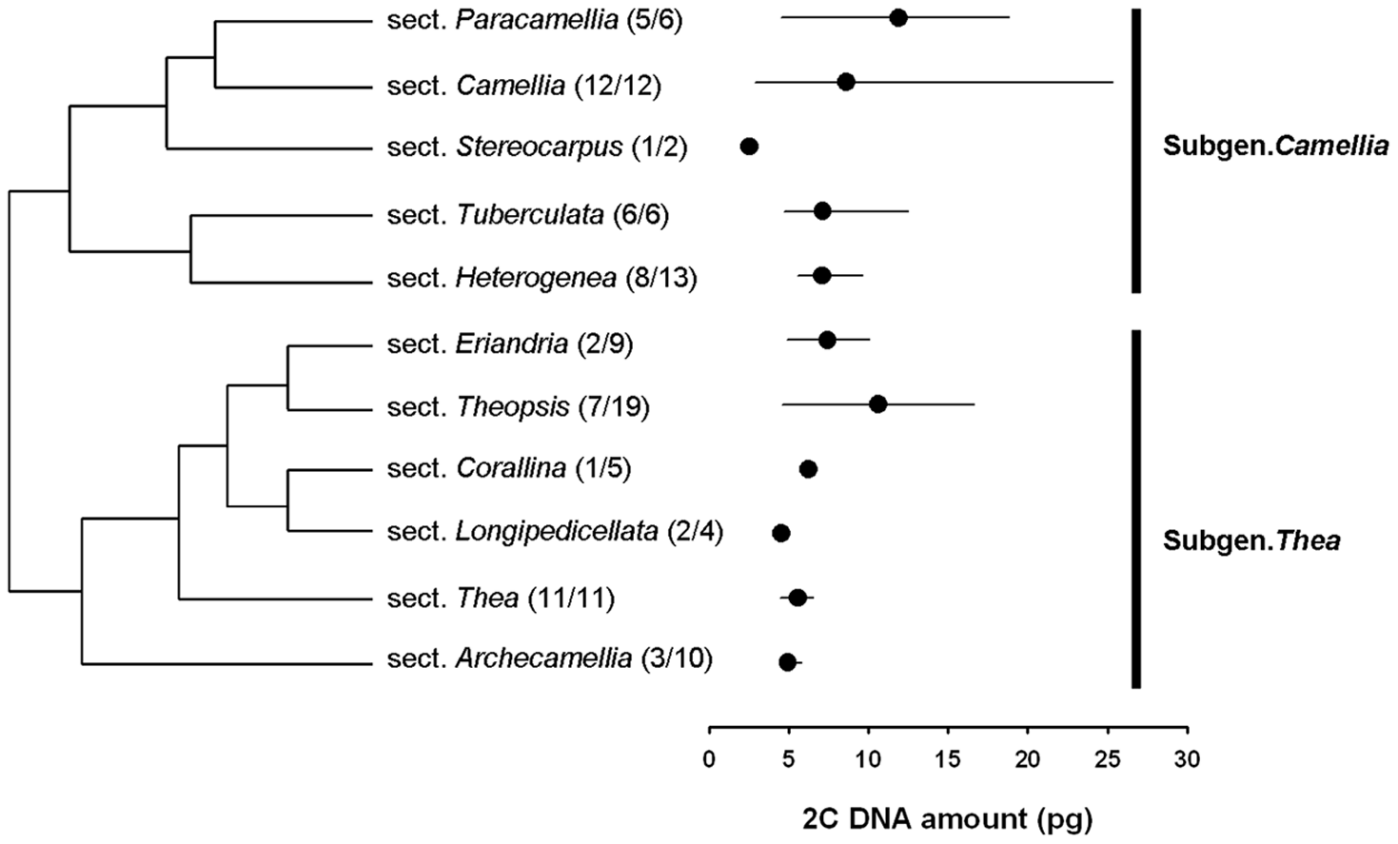
Nuclear DNA contents and evolutionary relationships among members of the genus *Camellia*. The indicated phylogenetic relationships of the genus were constructed by using morphological data and adopted from Min et al. [28]. The numbers in brackets for each section represent the number of species with the measured nuclear DNA content followed by the total number of species comprising the section. The mean 2C DNA amount is indicated by • for each section, while the range is shown as a line from the minimum to maximum 2C DNA amounts. The two subgenera recognized in *Camellia* are given on the right side of the figure.

Nuclear DNA contents were more extensively sampled and examined, in addition to the above-described sections of *Thea* and *Camellia*, for a total of 38 representative species from the 10 sections [28] or 13 sections [31] in the genus *Camellia* (Table 5). The chromosome numbers of those measured species which were adopted from previous studies and ploidy levels which were estimated based on DNA contents were showed in Table 5. The genus *Camellia* was phylogenetically split into the two subgenera, *Camellia* and *Thea* [28]. Superimposing 2C-values onto a phylogenetic tree provides an interpretation of the evolutionary direction(s) of genome size evolution in the genus *Camellia* (Fig. 6). Increases in DNA content have apparently occurred not only in the subgenus *Thea* but also in the subgenus *Camellia*. The subgenus *Camellia* apparently exhibited a larger DNA content variation (10.0-fold, 2C = 2.54–25.35 pg) probably due to the polyploidization than the subgenus *Thea*.

**Table 5:** Nuclear DNA amount of representing species in the genus *Camellia* estimated with flow cytometry.

Species (Min et al. 2010) [28]	Species (Chang and Ren, 1998) [31]	Chromosome Number (2n)	2C-value (pg)	SD	Estimation of Ploidy Levels
**sect. ** ***Paracamellia***	**sect. ** ***Oleifera***				
*C. fluviatilis* var. *megalantha*	*C. lanceoleosa*	NA*	4.53	0.469	2n = 2x
*C. gauchowensis*	*C. gauchowensis*	75	17.98	0.992	2n = 8x
*C. oleifera*	*C. oleifera*	30,45,90	17.47	0.970	2n = 8x
*C. sasanqua*	*C. sasanqua*	45–120	18.79	0.868	2n = 8x
**sect. ** ***Paracamellia***	**sect. ** ***Paracamellia***				
*C. brevistyla* var. *microphylla*	*C. microphylla*	30	5.48	0.325	2n = 2x
*C. grijsii*	*C. grijsii*	60	4.93	0.446	2n = 2x
*C. grijsii* var. *grijsii*	*C. yuhsienensis*	NA*	15.22	0.262	2n = 6x
*C. kissii* var. *confusa*	*C. confusa*	NA*	10.86	1.207	2n = 4x
**sect. ** ***Tuberculata***	**sect. ** ***Tuberculata***				
*C. anlungensis* var. *anlungensis*	*C. obovatifolia*	30	9.38	0.619	2n = 4x
*C. ilicifolia* var. *ilicifolia*	*C. rubimuricata*	30	5.36	0.439	2n = 2x
*C. parvimuricata* var. *hupehensis*	*C. hupehensis*	NA*	12.48	0.425	2n = 6x
*C. pyxidiacea* var. *rubituberculata*	*C. rubituberculata*	30	4.59	0.272	2n = 2x
*C. rhytidocarpa*	*C. rhytidocarpa*	NA*	4.75	0.299	2n = 2x
*C. tuberculata*	*C. tuberculata*	NA*	8.52	1.130	2n = 4x
**sect. ** ***Archecamellia***	**sect. ** ***Chrysantha***				
*C. huana*	*C. liberofilamenta*	30	5.79	0.233	2n = 2x
*C. impressinervis*	*C. impressinervis*	30	4.59	0.167	2n = 2x
*C. petelotii*	*C. nitidissima*	30	4.48	0.194	2n = 2x
**sect. ** ***Theopsis***	**sect. ** ***Theopsis***				
*C. costei*	*C. dubia*	30	12.25	0.772	2n = 6x
*C. crassipes*	*C. crassipes*	30	16.64	1.713	2n = 8x
*C. fraterna*	*C. fraterna*	90	12.71	0.439	2n = 6x
*C. rosthorniana*	*C. rotsthorniana*	NA*	4.61	0.237	2n = 2x
*C. synaptica* var. *synaptica*	*C. tsaii*	30	12.72	0.710	2n = 6x
*C. transarisanensis*	*C. handelii*	NA*	4.83	0.217	2n = 2x
**sect. ** ***Longipedicellatae***	**sect. ** ***Longissima***				
*C. longissima*	*C. longissima*	NA*	4.67	0.185	2n = 2x
**sect. ** ***Longipedicellatae***	**sect. ** ***Longipedicellatae***				
*C. longipedicellata*	*C. longipedicellata*	NA*	4.38	0.206	2n = 2x
**sect. ** ***Stereocarpus***	**sect. ** ***Luteoflora***				
*C. luteoflora*	*C. luteoflora*	30	2.54	0.209	2n = 2x
**sect. ** ***Eriandria***	**sect. ** ***Eriandria***				
*C. lawii*	*C. lawii*	30	4.86	0.275	2n = 2x
*C. salicifolia*	*C. salicifolia*	NA*	10.02	0.523	2n = 4x
**sect. ** ***Tuberculata***	**sect. ** ***Pseudocamellia***				
*C. tuberculata* var. *tuberculata*	*C. chungkingensis*	NA*	4.83	0.772	2n = 2x
**sect. ** ***Heterogenea***	**sect. ** ***Furfuracea***				
*C. crapnelliana*	*C. crapnelliana*	30	5.58	0.263	2n = 2x
*C. crapnelliana*	*C. gigantocarpa*	30	5.61	0.386	2n = 2x
*C. pubifurfuracea*	*C. pubifurfuracea*	NA*	6.77	0.274	2n = 2x
**sect. ** ***Heterogenea***	**sect. ** ***stereocarpus***				
*C. yunnanensis* var. *camellioides*	*C. liberistyloides*	NA*	5.85	0.197	2n = 2x
*C. yunnanensis*	*C. yunnanensis*	30	5.91	0.007	2n = 2x
**sect. ** ***Heterogenea***	**sect. ** ***Pseudocamellia***				
*C. yunnanensis* var. *camellioides*	*C. trichocarpa*	NA*	8.46	0.356	2n = 4x
**sect. ** ***Heterogenea***	**sect. ** ***Archecamellia***				
*C. granthamiana*	*C. albogigas*	60	8.98	0.202	2n = 4x
*C. granthamiana*	*C. granthamiana*	60	9.66	0.705	2n = 4x
**sect. ** ***Corallinae***	**sect. ** ***Thea***				
*C. pubicosta*	*C. pubicosta*	30	6.24	0.323	2n = 2x

*Z. mays* L. cv. B73 was employed as a standard. Chromosome numbers were adopted from previous studies and the index to Plant Chromosome Numbers (http://mobot.mobot.org/W2T/Search/ipch.html). All germplasms were collected from International *Camellia* Species Garden (ICSG).

* NA indicates that the information of chromosome number is not available.

## Discussion

### Performance of flow cytometry for the *Camellia* species

High content of cytosolic compounds in the tissues of plants like the *Camellia* species often attracts the attention to facilitate the selection of the most appropriate buffer [46]. In addition to releasing nuclei from intact cells, lysis buffers must ensure the stability of nuclei throughout the experiment, protect DNA from degradation and ease stoichiometric staining. We finally selected and employed an improved WPB isolation buffer in the flow cytometry, which was able to counteract the negative effects of tannic acid (TA) [41] and reliably provided excellent results with lower CV<5%. In the improved WPB isolation buffer, PVP was added to bind the phenolics kept in a reduced state [34] and thus suppressed the TA effect [41]. The antioxidant dithiothreitol, a substance that preserves chromatin integrity and minimizes stoichiometric errors in the DNA staining was also added in the experiments. Loureiro et al. [34] also confirmed that WPB is suitable for the analysis of problematic tissue or species. The explanation for our excellent results of this WPB buffer may be able to improve chromatin accessibility and ‘homogenizes’ chromatin structure, eliminating differences in staining intensity among nuclei with the same DNA content. The suitable plant tissues for flow cytometry should ideally contain rapidly dividing cell without substances that interfere with the experiment. In the eight investigated species of *Camellia*, comparisons of flow cytometry data obtained from the flowers, leaves and buds showed little discrepancy of DNA contents among different tissues. Accordingly, leaves were selected for the evaluation of DNA contents in the next experiments in the present study. In the leaves of *Camellia*, specialized cells often accumulate different phenolic compounds, such as tannins in particular, which may interfere with the flow cytometry [47], [48]. Because phenolic compounds and other oxypurines are known to bind with DNA, modify DNA-supercoiling, and form a complex with intercalating dye [49]. The experimental artifacts were observed in *Pinaceae* species [50], which was called as ‘tannic acid effect’ [40]. However, the opposite results were obtained in the nuclei of sunflower leaves isolated in Galbraith's buffer, despite increasing the variance of the peaks [14]. Other oxypurines and alkaloids could interfere with the phenolic effect [51]. For the tea tree, dye accessibility variations are likely to be the result of caffeine-chlorogenic acids (CGA) interactions, which is often rich in secondary metabolites [15]. In our experiment, we found that *C. sinensis* var. *assamica* brought impurity into the solution showing with low intensity peaks, and thus led to the slight variation of PI fluorescence of maize when they were treated simultaneously (Fig. 1). The competition between PI and phenolic compound is thus expected, resulting in a drop in PI accessibility to DNA. Nevertheless, the impact of secondary metabolite on the fluorescence of *Camellia* nuclei is slight with a 0.1 pg/2C discrepancy so that it is enough to gain credible estimates of *Camellia* DNA content by flow cytometry.

In this study, maize (*Z. mays* L. cv. B73) with a DNA content of 1C = 2.35 pg was used as the standard to estimate nuclear DNA contents of the *Camellia* representative sections and species. An ideal scenario is to use the plant species whose genome has been completely sequenced as a reference standard and thus the genome size may accurately be determined. However, up to date, there are not any genomes have been fully sequenced, given the assembly difficulties of repeat sequences and particularly heterochromatin regions in telomeres and centromere that cannot be easily sequenced. While it is certainly true that the C-values assumed for standards can vary depending on a number of factors [52], [53], [54], this study selected maize as a reference since genome size of the species has been roughly determined comparing with numerous plants without genome sequences available [35]. Among the other sequenced plants, the estimated genome size of maize (∼2300 Mb) is comparatively close to the tea tree, and thus may be suitable to serve as a standard and obtain a relatively reliable estimation of the *Camellia* species.

### Genome size estimation of *C. sinensis* var. *assamica* and its intraspecific variation

As *C. sinensis* var. *assamica* was reported as a diploid (2n = 30) [55], karyological uniformity and the characteristic of all cultivars of the species make it a suitable example to study intraspecific genome size variation. The 2C DNA content varied 1.1-fold among 17 cultivars of *C. sinensis* var. *assamica*, indicated that there was a low level of intraspecific variation of the genome size among the measured cultivars of *C. sinensis* var. *assamica*. Despite the fact that genome size is more likely constant at species level, intraspecific variation was indeed observed and characterized in various plant species [19]. Genome size variation is common among congeneric species [56], subspecies [57] and populations [58], [59]. This is particularly noticeable in the species with extensive geographic distribution that shows high morphological differentiation and includes several subspecific categories. In the absence of polyploidy and changes in chromosome number [60], significant variations in genome size could be due either to fluctuations within highly repetitive DNA such as retrotransposons [27], [61] or to structural rearrangements such as small amplifications and deletions at the individual chromosomal level [62]. In addition, the simultaneous presence of ‘phenolics-alkaloids’ could lead to interactions and slight intraspecific variations in nuclear DNA content of *C. sinensis* var. *assamica* [15]. In this study, our results showed that there was a lack of latitudinal effect on intraspecific variation in genome size of the examined cultivars of *C. sinensis* var. *assamica*.

Based on the mean DNA content of all the measured cultivars (1C = 3.01 pg), the genome size of *C. sinensis* var. *assamica* was estimated to be 2940 Mb by using 1 pg DNA = 978 Mb [42]. Our result apparently conflicted with a previous estimation that genome size of *C. sinensis* was estimated to be 4000 Mb [63]. The discrepancy might originate from RNA digestion by RNase and fluorescent-dye which were simultaneously performed [63], resulting in an overestimation due to the interference of RNA binding with PI. Note that this is the first effort to estimate genome size of the *Camellia* species by using a standard with which genome size is better known from the sequenced genome. Thus, another likely explanation is that the internal standards formerly employed were based on uninsurable estimates of genome size from organisms (e.g. soybean and wheat) yet to be sequenced.

### Interspecific genome size variation in the genus *Camellia*

The DNA contents of interspecific variation (1.5-fold) in the section *Thea*, as expected, was somewhat larger than intraspecific variation (1.1-fold) among the representative cultivars of *C. sinensis* var. *assamica*. Apparently, our estimates of DNA ploidy (2n = 2x) based on DNA contents of these measured species were confirmed by conventional chromosome counting (2n = 30). Given the absence of polyploidization and changes in chromosome number in the section *Thea* [43], [55], it is likely that the variations in genome size among different species might be caused by fluctuations within highly repetitive DNA such as retrotransposons [27], [61] and structural rearrangements [62]. The present study revealed that, in spite of slight variations, nuclear DNA contents were not randomly distributed and appeared largely conserved across the majority of the species under investigation. There were different opinions with regard to taxonomic treatment on *C. pubicosta*, which was classified into the section *Thea* by Chang et al. [31] but was recently treated as a member of the section *Corallinae* by Min et al. [28]. Considering that differences within related species were much fewer than those irrelevant species [11], the finding suggests that *C. pubicosta* and *C. pubescens* might have a distant relationship at least in term of genome size evolution and thus require to further study the taxonomic treatment on *C. pubicosta*.

The section *Camellia* is a taxonomically complicated group of plants that is substantially influenced by frequent interspecific hybridization and polyploidization [28]. The mean 2C-value of the section *Camellia* species was 8.61 pg, with a 5.78 standard deviation, larger than that of the section *Thea* (5.60 pg) with a 0.63 standard deviation. While levels of polyploidy used in this study were based on previous chromosome counts, the results should always be designated as “DNA ploidy” or “DNA aneuploidy” as some chromosome counts are lacking [64]. Only with the aid of FCM, has it been possible to reliably assess ploidy distribution at various spatial scales, interactions among cytotypes, and evolutionary processes in diploid-polyploid sympatric populations [65], [66]. Based on the estimation of DNA contents, DNA ploidy levels for the 53 studied species were approximately determined (Table 4; Fig. 4). We inferred that DNA ploidy levels of the studied species ranged largely including 2n = 2x, 4x, 6x, 8x, 10x and 12x when an average estimation of ∼4.91 pg was applied at the diploid level. Although ploidy estimation by cytometric techniques is generally considered to be a trivial task, some precautions should be taken during data interpretation [64]. For example, there is a possibility that changes in genome size independent of polyploidy could be taking place within the genus *Camellia*. Our estimates of different DNA ploidy levels of these measured species should be further confirmed by conventional chromosome counting. Chromosome counts (2n = 30, 45, 60, 90, 120) [55], [67], [68] and our estimates of different DNA ploidy levels (2n = 2x–12x) of these measured species (Table 4) together indicate that the polyploidization and interspecific hybridization may mainly account for the patterns of large DNA content variation in this section. It is the polyploidization that has made the evolution of DNA content within the section appears phasic variation rather than gradual. In addition, our results showed that DNA content varied among different diploid species, suggesting that there may be the other factors causing the difference of genome size in this section. The most likely explanation is the varied extent of amplification of repeat sequences [4], [60] occurred in different species and possible hybridization between closely related taxa [58]. We further showed that DNA contents were mainly conserved among closely related species and its variation is nearly consistent to evolutionary relationships of the section *Camellia* species, as indicated by molecular phylogenetic evidence [45]. Accordingly, our results further support that nuclear DNA content has a predictive value for inferring evolutionary relationships [32]. While genome size data can help to understand evolutionary relationships, there are many cases where the variation between species is not at all helpful as one can get big differences in genome size between closely related species.

### Genome size evolution of the genus *Camellia*

Many studies on a currently unresolved question on the variation of DNA contents from a phylogenetic perspective suggested that the evolutionary direction(s) of DNA content in plants could increase [27], decrease [22], [57], or exhibit a bio-directional dynamic [1]. The genus *Camellia* was phylogenetically split into the two subgenera, *Camellia* and *Thea* [28]. Increases in DNA content have apparently occurred not only in the subgenus *Thea* but also in the subgenus *Camellia*. Our results suggested that the ‘increase’ hypothesis for genome size evolution may hold true in the genus *Camellia*. There are a small number of reductions of DNA content in certain lineages might due to an incomplete sampling. We found that the diploid species account for a large percentage of those measured species, representing in all those sampled sections. It seems likely that the speciation occurred among different sections of the genus earlier than polyploidization events, leading to that all sections contained diploids in addition to polyploidy species. It is clear that polyploidization occurred more frequently in the recently diverged sections (e.g. sections *Paracamellia* and *Camellia*, MTS) than other sections (e.g. section *Stereocarpus*, MTS) in the two subgenera. In addition, the majority of the 26 studied *Camellia* species are hexaploid. It may be inferred that the polyploidization may main lead evolutionary direction of the genus *Camellia*, which is consistent to the previous study [69]. Moreover, artificial selection might have played an ineligible role in genome size evolution of the genus *Camellia* on account of the advantages and ornamental value of polyploidy with large flowers. With the hope of outlining a full picture of genome size variation and evolution of the genus *Camellia*, the future work is needed to investigate phylogenetic relationships, karyotypes and genome sizes of other undetermined species.

## References

[1] Soltis DE, Soltis PS, Bennett MD, Leitch IJ (2003) Evolution of genome size in the angiosperms. Am J Bot 90: 1596–1603. 21653334 10.3732/ajb.90.11.1596

[2] Chase MW, Hanson L, Albert VA, Whitten WM, Williams NH (2005) Life history evolution and genome size in subtribe *Oncidiinae* (Orchidaceae). Ann Bot 95: 191–199. 15596466 10.1093/aob/mci012 PMC4246717

[3] Beaulieu JM, Moles AT, Leitch IJ, Bennett MD, Dickie JB, et al (2007) Correlated evolution of genome size and seed mass. New Phytol 173: 422–437. 17204088 10.1111/j.1469-8137.2006.01919.x

[4] Wakamiya I, Newton R, Johnston JS, Price HJ (1993) Genome size and environmental factors in *Pinus* . Am J Bot 80: 1235–1241.

[5] Lee CE (2002) Evolutionary genetics of invasive species. Trend Ecol Evol 17: 386–391.

[6] Bennett MD, Leitch IJ (2011) Nuclear DNA amounts in angiosperms: targets, trends and tomorrow. Ann Bot 107: 467–590. 21257716 10.1093/aob/mcq258 PMC3043933

[7] Grover CE, Yu Y, Wing RA, Paterson AH, Wendel JF (2008) A phylogenetic analysis of indel dynamic in the cotton genus. Mol Biol Evol 7: 1415–1418. 10.1093/molbev/msn085 18400789

[8] Price HJ (1988) Nuclear DNA content variation within angiosperm species. Evol Trend Plant 2: 53–60.

[9] Leong-Skornickova J, Sida O, Jarolimova V, Sabu M, Fer T, et al (2007) Chromosome numbers and genome size variation in Indian species of *Curcuma* (Zingiberaceae). Ann Bot 100: 505–526. 17686760 10.1093/aob/mcm144 PMC2533610

[10] Laurie DA, Bennett MD (1985) Nuclear DNA content in the genera *Zea* and *Sorghum*. Intergeneric, interspecific and intraspecific variation. Heredity 55: 307–313.

[11] Cullis CA (2005) Mechanisms and control of rapid genomic changes in flax. Ann Bot 95: 201–206. 15596467 10.1093/aob/mci013 PMC4246718

[12] Greilhuber J (2005) Intraspecific variation in genome size in angiosperms: identifying its existence. Ann Bot 95: 91–98. 15596458 10.1093/aob/mci004 PMC4246709

[13] Greilhuber J (1998) Intraspecific variation in genome size: a critical reassessment. Ann Bot 82: 27–35.

[14] Price HJ, Hodnett G, Johnston JS (2000) Sunflower (*Helianthus annuus*) leaves contain compounds that reduce nuclear propidium iodide fluorescence. Ann Bot 86: 929–934.

[15] Noirot M, Barre P, Duperrayy C, Louran J, Hamon S (2003) a. Effects of caffeine and chlorogenic acid on propidium iodide accessibility to DNA: consequences on genome size evaluation in coffee tree. Ann Bot 92: 259–264. 12876189 10.1093/aob/mcg139 PMC4243661

[16] Lysák MA, Rostková A, Dixon JM, Rossi G, Doležel J (2000) Limited genome size variation in *Sesleria albicans* . Ann Bot 86: 399–403.

[17] Le Thierrry d'Ennequin M, Panaud O, Brown S, Siljak-Yakovlev A, Sarr A (1998) First evaluation of DNA content in *Settaria* genus by flow cytometry. J Hered 89: 556–559.

[18] Ellul P, Boscaiu M, Vicente O, Moreno V, Roselló JA (2002) Intra- and interspecific variation in DNA content in *Cistus* (Cistaceae). Ann Bot 90: 345–351. 12234146 10.1093/aob/mcf194 PMC4240394

[19] Moscone EA, Baranyi M, Ebert I, Greilhuber J, Ehrendorfer F, et al (2003) Analysis of nuclear DNA content in *Capsicum* (Solanaceae) by flow cytometry and Feulgen densitometry. Ann Bot 92: 21–29. 12824068 10.1093/aob/mcg105 PMC4243630

[20] Baranyi M, Greilhuber J (1995) Flow cytometric analysis of genome size variation in cultivated and wild *Pisum sativum* (Fabaceae). Plant Syst Evol 194: 231–239.

[21] Bennett MD, Johnston S, Hodnett GL, Price HJ (2000) *Allium cepa* L. cultivars from four continents compared by flow cytometry show nuclear DNA constancy. Ann Bot 85: 351–357.

[22] Wendel JF, Cronn RC, Johnston JS, Price HJ (2002) Feast and famine in plant genomes. Genetica 115: 37–47. 12188047 10.1023/a:1016020030189

[23] Barakat A, Carels N, Bernardi G (1997) The distribution of genes in the genomes of *Gramineae* . P Natl Acad Sci USA 94: 6857–6861. 10.1073/pnas.94.13.6857 PMC21249 9192656

[24] Grover CE, Wendel JF (2010) Recent insights into mechanisms of genome size change in plants. J Bot doi:10.1155/2010/382732.

[25] Piegu B, Guyot R, Picault N, Roulin A, Saniyal A, et al (2006) Doubling genome size without polyploidization: dynamics of retrotransposition-driven genomic expansions in *Oryza australiensis*, a wild relative of rice. Genome Res 16: 1262–1269. 16963705 10.1101/gr.5290206 PMC1581435

[26] Wicker T, Keller B (2007) Genome-wide comparative analysis of copia retrotransposons in *Triticeae*, rice, and *Arabidopsis* reveals conserved ancient evolutionary lineages and distinct dynamics of individual copia families. Genome Res 17: 1072–1081. 17556529 10.1101/gr.6214107 PMC1899118

[27] Bennetzen J, Ma J, Devos K (2005) Mechanisms of recent genome size variation in flowering plants. Ann Bot 95: 127–132. 15596462 10.1093/aob/mci008 PMC4246713

[28] Min TL, Wu ZY, Li DZ, Hong DY, Zhang XC, et al.. (2010) Flora of China. Science Press. pp. 366–478.

[29] Krahulcová A, Krahulec F (2000) Offspring diversity in *Hieracium* subgen. *Pilosella* (Asteraceae): new cytotypes from hybridization experiments and from open pollination. Fragm Flor Geobot 45: 239–255.

[30] Kamemoto H (1987) Genome breeding in Dendrobium orchids. In: The breeding of horticultural crops Chang WN, Opena RT, editors. Taipei: FFTC 35: 182–188.

[31] Chang HD, Ren SX (1998) Flora of China. Science Press. Tomus 49 (3) 1–251.

[32] Suda J, Krahulcová A, Trávnícek P, Rosenbaumová R, Peckert T, et al (2007) Genome size variation and species relationships in *Hieracium* sub-genus *Pilosella* (Asteraceae) as inferred by flow cytometry. Ann Bot 100: 1323–1355. 17921526 10.1093/aob/mcm218 PMC2759259

[33] Galbraith DW, Harkins KR, Maddox JM, Ayres NM, Sharma DP, et al (1983) Rapid flow cytometric analysis of the cell-cycle in intact plant-tissues. Science 220: 1049–1051. 17754551 10.1126/science.220.4601.1049

[34] Loureiro J, Rodriguez E, Doležel J, Santos C (2007) Two new nuclear isolation buffers for plant DNA flow cytometry: a test with 37 species. Ann Bot 100: 875–888. 17684025 10.1093/aob/mcm152 PMC2749623

[35] Schnable PS, Ware D, Fulton RS, Stein JC, Wei FS, et al (2009) The B73 maize genome: complexity, diversity and dynamics. Science 326: 1112–1115. 19965430 10.1126/science.1178534

[36] Doležel J, Binarová P, Lucretti S (1989) Analysis of nuclear DNA content in plant cells by flow cytometry. Biol Plantarum 31: 113–120.

[37] Otto F (1992) Preparation and staining of cells for high-resolution DNA analysis. In: Radbruch A, editor. Flow cytometry and cell sorting. Berlin: Springer-Verlag, pp. 101–104.

[38] Doležel J, Göhde W (1995) Sex determination in dioecious plants *Melandrium album* and *M. rubrum* using high-resolution flow cytometry. Cytometry 19: 103–106. 7743889 10.1002/cyto.990190203

[39] Pfosser M, Amon A, Lelley T, Heberle-Bors E (1995) Evaluation of sensitivity of flow cytometry in detecting aneuploidy in wheat using disomic and ditelosomic wheat-rye addition lines. Cytometry 21: 387–393. 8608738 10.1002/cyto.990210412

[40] Loureiro J, Rodriguez E, Doležel J, Santos C (2006) a. Flow cytometric and microscopic analysis of the effect of tannic acid on plant nuclei and estimation of DNA content. Ann Bot 98: 515–527. 16820406 10.1093/aob/mcl140 PMC2803573

[41] Loureiro J, Rodriguez E, Doležel J, Santos C (2006) b. Comparison of four nuclear isolation buffers for plant DNA flow cytometry. Ann Bot 98: 679–689. 16820407 10.1093/aob/mcl141 PMC2803574

[42] Doležel J, Bartoš J, Voglmayr H, Greilhuber J (2003) Nuclear DNA content and genome size of trout and human. Cytometry A 5: 127–128. 10.1002/cyto.a.10013 12541287

[43] Li GT, Liang T, Zhang RQ, Zhang ML (2007) Study of the karyotypes of some species of section *Thea* . J Tea Bus 1: 25–28.

[44] Chen L, Yamaguchi S, Wang PS, Xu M, Song WX, et al (2002) Genetic polymorphism and molecular phylogeny analysis of section *Thea* based on RAPD markers. J Tea Sci 22: 19–24.

[45] Tian M, Li JY, Ni S, Fan ZQ, Li XL (2008) Phylogenetic study on section *Camellia* based on ITS sequences data. Acta Hort Sin 35: 1685–1688.

[46] Kuo J, McComb AJ (1989) Seagrass taxonomy, structure and development. In: Larkum AWD, McComb A, Shepherd SA, editors. Biology of Seagrasses. Elsevier, Amsterdam. pp. 112–156.

[47] Greilhuber J (1988) ‘Self-tanning’-a new and important source of stoichiometric error in cytophotometric determination of nuclear DNA content in plants. Plant Syst Evol 158: 87–96.

[48] Greilhuber J (1986) Severly distorted Feulgen DNA amounts in *Pinus* (Coniferophytina) after nonadditive fixations as a result of meristematic self-tanning with vacuole contents. Can J Genet Cytol 28: 409–415.

[49] Traganos F, Kapuscinski J, Darzynkiewicz Z (1991) Caffeine modulates the effects of DNA-intercalating drugs *in vitro* a flow cytometric and spectrophotometric analysis of caffeine interaction with novantrone, doxorubicine, ellipticine and the doxorubicne analogue AD198. Cancer Res 51: 3682–3689. 2065324

[50] Greilhuber J (1986) Severly distorted Feulgen DNA amounts in *Pinus* (Coniferophytina) after nonadditive fixations as a result of meristematic self-tanning with vacuole contents. Can J Genet Cytol 28: 409–415.

[51] Noirot M, Poncet V, Barre P, Hamon P, Hamon S, et al (2003) b. Genome size variations in diploid African coffea species. Ann Bot 92: 709–714. 14573524 10.1093/aob/mcg183 PMC4244848

[52] Dolezel J, Greilhuber J (2010) Nuclear genome size: are we getting closer? Cytometry 77A: 635–642. 10.1002/cyto.a.20915 20583277

[53] Bennett MD, Leitch IJ (2011) Nuclear DNA amounts in angiosperms: targets, trends and tomorrow. Ann Bot 107: 467–509. 21257716 10.1093/aob/mcq258 PMC3043933

[54] Suda J, Leitch IJ (2010) The quest for suitable reference standards in genome size research. Cytometry 77A: 717–720. 10.1002/cyto.a.20907 20653010

[55] Gu ZJ, Sun XF (1997) A karyomorphological study of seventeen species of Chinese *Camellia* . Acta Bot Yunn 19: 159–170.

[56] Price HJ (1976) Evolution of DNA content in higher plants. Bot Review 42: 27–52.

[57] Price HJ, Dillon SL, Hodnett G, Rooney WL, Ross L, et al (2005) Genome evolution in the genus *Sorghum* (Poaceae). Ann Bot 95: 219–227. 15596469 10.1093/aob/mci015 PMC4246720

[58] Hall SE, Dvorak WS, Johnston JS, Price HJ, Willians CG (2000) Flow cytometric analysis of DNA content for tropical and temperate new world pines. Ann Bot 86: 1081–1086.

[59] Šmarda P, Bureš P (2006) Intraspecific DNA content variability in *Festuca pallens* on different geographical scales and ploidy levels. Ann Bot 98: 665–678. 16868002 10.1093/aob/mcl150 PMC2803578

[60] Ohri D, Khoshoo TN (1986) Genome size in gymnosperms. Plant Syst Evol 153: 119–132.

[61] Šmarda P, Bureš P (2010) Understanding intraspecific variation in genome size in plants. Preslia 82: 41–61.

[62] Williams RR, Broad S, Sheer D, Ragoussis J (2002) Subchromosomal positioning of the epidermal differentiation complex (EDC) in keratinocyte and lymphoblast interphase nuclei. Exp Cell Res 272: 163–175. 11777341 10.1006/excr.2001.5400

[63] Tanaka J, Taniguchi F (2006) Estimation of the genome size of Tea (*Camellia sinensis*), Camellia (*C. japonica*), and their interspecific hybrids by flow cytometry. J Tea Res 101: 1–7.

[64] Soda J, Krahulcová A, Trávnícek P, Krahulec F (2006) Ploidy level versus DNA ploidy level: an appeal for consistent terminology. Taxon 55: 447–450.

[65] Baack EJ (2004) Cytotype segregation on regional and microgeographic scales in snow buttercups (*Ranunculus adoneus*: Ranunculaceae). Amer J Bot 91: 1783–1788. 21652325 10.3732/ajb.91.11.1783

[66] Husband BC, Sabara HA (2004) Reproductive isolation between autotetraploids and their diploid progenitors in fireweed, *Chamerion angustifolium* (Onagraceae). New Phytol 161: 701–711. 10.1046/j.1469-8137.2004.00998.x 33873724

[67] Li GT, Liang T (1990) The chromosome counts and karyomorphological study of *Camellia* . Guihaia 10: 127–138.

[68] Li GT (2001) New advance in karyotype studies of genus *Camellia* . Chinese Wild Plant Res 20: 9–14.

[69] Chang HD (1981) Systemic research on Camellia. Zhongshan University Press. pp. 52–84.

